# Psychometric Evaluation and Comparison of Two Gaming Disorder Measures Derived From the DSM-5 and ICD-11 Frameworks

**DOI:** 10.3389/fpsyt.2020.577366

**Published:** 2020-12-17

**Authors:** Hsin-Yi Wang, Cecilia Cheng

**Affiliations:** Department of Psychology, The University of Hong Kong, Pokfulam, Hong Kong

**Keywords:** internet gaming disorder, online gaming, gaming addiction, behavioral addiction, scale validation, measurement invariance, psychometric comparison, psychometric properties

## Abstract

Gaming disorder was listed as a condition for further study in the 5th edition of the *Diagnostic and Statistical Manual of Mental Disorders* (DSM-5) in 2013, and measures of the disorder have mushroomed in the years since. The Gaming Disorder Test (GDT) was developed after gaming disorder was officially included in the 11th Revision of the International Classification of Diseases (ICD-11) in 2018. However, it remains unknown whether the GDT, which is based on the ICD-11 framework, is psychometrically similar to or different from the popular nine-item Internet Gaming Disorder Scale-Short Form (IGDS9-SF) based on the DSM-5 framework. To address this important but unexplored issue, the present study evaluated and compared the psychometric properties of the GDT and IGDS9-SF in a sample of 544 adult gamers (56.2% men; mean age = 28.8, *SD* = 8.55). The results revealed both measures to have good reliability, structural validity, and criterion validity, with the exception of one IGDS9-SF item with a low factor loading. Moreover, the IGDS9-SF exhibited scalar measurement invariance for gender and age but only partial metric invariance for employment status, whereas the GDT exhibited scalar measurement invariance for all three demographic characteristics. Finally, the GDT displayed incremental validity over the IGDS9-SF in explaining gaming time, but not social anxiety and depressive symptoms. This study thus contributes to the literature by comparing measures derived from distinct gaming disorder diagnostic frameworks empirically. Recommendations for the selection of gaming disorder measures by researchers and practitioners are discussed.

## Introduction

Video gaming has become an integral part of life for many players, but gaming can become problematic if it interferes with psychosocial functioning [e.g., (1, 2)]. Reflecting widespread concerns over problematic gaming, Internet gaming disorder was listed as a condition for further study in the 5th edition of the *Diagnostic and Statistical Manual of Mental Disorder* [DSM-5; (3)]. The DSM-5 framework comprises nine criteria: 1) preoccupation with gaming; 2) withdrawal symptoms when gaming is not accessible; 3) increasing amounts of time spent on gaming; 4) unsuccessful attempts to control gaming; 5) loss of interest in other hobbies or activities; 6) continued excessive gaming despite knowledge of the undesirable consequences; 7) deceiving others regarding the amount of gaming; 8) use of gaming to escape from unpleasant moods; and 9) losing significant interpersonal relationships due to gaming. Although the DSM-5 labels the problem “Internet gaming disorder,” it states that the disorder also involves non-Internet or offline games (3). Since the DSM-5 framework's proposal, scholars have advocated for the establishment of standardized assessment tools based on the nine DSM-5 criteria (4, 5). In response, researchers have adopted the DSM-5 framework to develop measures of gaming disorder [e.g., (6)], with one of the most popular measure being the nine-item Internet Gaming Disorder Scale-Short Form [IGDS9-SF; (7)]. The IGDS9-SF has been widely adopted and validated in a range of cultural regions such as Australia, Hong Kong, Portugal, and Turkey [e.g., (8–11)].

More recently, gaming disorder was officially included as a mental health disorder in the 11th version of the International Classification of Diseases [ICD-11 (12)] to facilitate further research and the formulation of social policy. The ICD-11 framework consists of four criteria: (1) impaired control over gaming; (2) increasing priority given to gaming as gaming takes precedence over other interests; (3) the continuation of gaming despite knowledge of the undesirable consequences, and (4) the problematic gaming behavior has led to significant disruptions to major life domains (e.g., interpersonal, job/academic performance) that last for at least 12 months. The Gaming Disorder Test [GDT (2)] was developed based on this framework, with the measure validated in China and the United Kingdom.

Apparently, the two clusters of measures differ in certain ways because they are constructed from frameworks with a distinct set of criteria. For example, the more concise ICD-11 framework places greater emphasis on the functional impairment aspect of gaming disorder (13). For instance, past studies have identified several symptoms included in the ICD-11 framework (i.e., “loss of control” and “giving up other activities”) to make strong contribution in identifying gaming disorder [e.g., (13, 14)]. In contrast, the more comprehensive DSM-5 framework encompasses a wider variety of cognitive and behavioral manifestations of the disorder.

A recent study has tested validated measures constructed from both frameworks, and the findings showed that the two measures yielded comparable prevalence rates and similar patterns of associations with mental health indicators (15). The present study extended their work by adopting a more naunced approach to scrutinize whether there are differences in psychometric properties among measures derived from distinct frameworks. Specifically, this study compares several types of validity of the newly developed GDT (2) and the widely adopted IGDS9-SF (7). We conducted four tests for evaluating the fundamental psychometric properties of these two measures, including structural validity, criterion validity, concurrent validity, and reliability analysis.

We further scrutinized whether the GDT and IGDS9-SF have measurement invariance, which refers to the statistical property whereby respondents reporting the same scores tend to exhibit an identical level of the underlying trait (16). As gaming disorder measures are often used in large-scale population surveys with heterogeneous community samples [e.g., (17–19)], measurement invariance is essential for detecting the influence of demographic characteristics in between-group comparisons.

In this study, we examined the measurement invariance properties across three demographic characteristics^1^: (1) gender (men vs. women), (2) age (younger vs. older players), and (3) employment status (students, full-time workers, vs. non-full-time workers). Potential variations in gender and age were tested because studies have indicated that gaming disorder tends to be more prevalent among male players and younger players [e.g., (24–28)]. Potential variations in employment status were tested because studies have revealed that student players are more likely to be active gamers than those in employment, and the employed players are more likely than students to purchase in-game items (29).

Moreover, incremental validity is another important property, particularly for evaluating the performance of new assessment tools relative to existing ones [e.g., (30, 31)]. As the IGDS9-SF has consistently been shown to account for the variance in such mental health-related criterion measures as depressive and social anxiety symptoms [e.g., (32, 33)], as well as gaming time [e.g., (10, 34)], it is important to test whether the new GDT can explain the variance in these criterion measures beyond the contribution made by the existing IGDS9-SF. Such findings can inform researchers and practitioners the unique contribution of the newly developed GDT, and such information is useful for assisting their decisions regarding whether to include the new or existing measures in their protocol.

## Materials and Methods

### Participants and Procedures

Participants were recruited from Prolific Academic, because studies have reported this online participant pool to be viable and valid for academic research; moreover, the participants recruited from this platform were found to be more diverse and less dishonest compared to other similar online platforms (35, 36). Our compensation rate ($1 for 10 min) adhered to the regulations of Prolific Academic. Eligible participants were adults (i.e., aged over 18) who responded “yes” to the screening question “Have you played any video game in the past 12 months?” To ensure good data quality, the participants were screened based on their reputation on the survey platform (i.e., >95% approval rating). The data collection process was conducted via an online survey hosted on Qualtrics. The present protocol received prior ethical approval from the institutional review board of the authors' university.

We recruited 544 participants from 34 countries. Most were from Europe (41.3%) and North America (40.5%), while the remaining were from Asia (12.1%) and other regions (9.3%). More than half (56.2%) were men, and the average age of the sample was 28.8 (*SD* = 8.55, range = 18–62). Roughly one-third (32.2%) reported themselves as current students, 40.4% as full-time workers, and 27.4% as non-full-time workers (i.e., working part-time, unemployed, or working in an unpaid position). For the present sample, the self-reported weekly gaming time was 19.9 hours (*SD* = 14.9) for general gaming and 13.2 h for online gaming (*SD* = 12.8).

### Measures

The Gaming Disorder Test [GDT (2)] was adopted to measure gaming disorder based on the ICD-11 framework. This scale includes four items to examine recurrent gaming over a 12-month period, regardless of the mode of gaming (e.g., online or offline) or the device used for playing (e.g., consoles, mobile devices, or personal computers). Participants were asked to rate all items on a 5-point Likert scale, ranging from 1 (never) to 5 (very often).

The nine-item Internet Gaming Disorder Scale-Short Form [IGDS9-SF; (7)] was selected to assess gaming disorder based on the DSM-5 framework. This instrument includes nine items that tap the severity of gaming disorder symptoms by evaluating gaming activities occurring over the past 12 months. Each item was measured on a 5-point Likert scale, ranging from 1 (*never*) to 5 (*very often*).

The short form of the Social Anxiety Interaction Scale [SIAS-6 (37)] was chosen to measure social anxiety symptoms. This scale adopts a unidimensional measurement approach to evaluate social apprehension based on six items. Participants were instructed to rate each item on a 5-point Likert scale, ranging from 1 (*not at all characteristic or true of me*) to 5 (*extremely characteristic or true of me*). The SIAS-6 was found to be reliable in this study (Cronbach's α = 0.88).

The short form of the Center for Epidemiological Studies Depression Scale [CES-D 10 (38)] was used to evaluate depressive symptoms. This unidimensional, non-clinical measure is comprised of 10 items that examined the frequency and duration of various symptoms of depression. Each item was measured in a 4-point Likert scale, ranging from 1 (*rarely or none of the time*) to 4 (*most or all of the time*). The CES-D had acceptable reliability in our study (Cronbach's α = 0.88).

The amount of gaming was assessed with two items asking how many hours per day, on average, participants played video games online and offline, respectively. Their weekly gaming time was calculated by multiplying the daily gaming time by 7 for each mode of video gaming.

Participants were also asked to report their age, gender, and employment status. For employment status, five options were provided: (1) student, (2) full-time worker, (3) part-time worker, (4) unemployed, and (5) not in paid work. To facilitate further analysis, the employment status is further recoded as a three-level construct, including student, full-time worker, and non-full-time worker, with the level of non-full-time worker comprising participants who were currently working part time, unemployed, and not in paid work.

### Statistical Analysis

All statistical analyses were performed with R-Studio version 3.4.1 (RStudio Team, Boston, MA) and SPSS version 23 (IBM Corp., Armonk, NY). The internal consistency of the measures was assessed using both Cronbach's alpha and McDonald's omega. McDonald's omega was obtained from the psych package for R [version 2.0.9 (39)]. Associations among the study variables were examined using Pearson zero-order correlation analysis. Confirmatory factor analysis (CFA) and multigroup CFA were performed using the lavaan package for R [version 5.20 (40)].

## Results

### Psychometric Property Evaluation

#### Reliability and Structural Validity

Both the GDT and IGDS9-SF were found to be reliable (Cronbach's alphas = 0.86 and 0.82, and McDonald's omega = 0.86 and.83, respectively). CFA was conducted for assessing structural validity. To evaluate model fit, we adopted the widely adopted criteria: CFI > 0.095, TLI > 0.095, RMSEA < 0.06, SRMR < 0.08 (41). As the scale items for both measures have five response options, the diagonally weighted least-squares method (DWLS) was used to estimate the model because of its suitability for CFA with ordered categorical variables. In addition, the issues of non-normality were not pertinent for our analyses, because the DWLS method was utilized. For the GDT, the CFA model showed a good fit to the data, as indicated by all five model fit indices: χ^2^ = 5.646, CFI = 0.991, TLI = 0.992, RMSEA = 0.017, and SRMR = 0.016. Moreover, all of the factor loadings were statistically significant, and no factor loading for an individual item was below 0.70 (see the upper panel of Table 1). Hence, these findings provide robust support for the structural validity of the GDT.

**Table 1:** Descriptive statistics and factor loadings derived from confirmatory factor analytical model for the two gaming disorder measures.

**Item**	***M***	***SD***	**Standardized factor loading**
**Gaming Disorder Test [GDT; (****2****)]**			
1. I have had difficulties controlling my gaming activity.	2.02	0.02	0.83
2. I have given increasing priority to gaming over other life interests and daily activities.	2.32	1.17	0.77
3. I have continued gaming despite the occurrence of negative consequences.	2.11	1.16	0.87
4. I have experienced significant problems in life (e.g., personal, family, social, education, occupational) due to the severity of my gaming behavior.	1.59	0.91	0.80
**Nine-item short form of the Internet Gaming Disorder Scale (IGDS9-SF; 7)**			
1. Do you feel preoccupied with your gaming behavior? (Some examples: Do you think about previous gaming activity or anticipate the next gaming session? Do you think gaming has become the dominant activity in your daily life?)	2.41	1.13	0.63
2. Do you feel more irritability, anxiety or even sadness when you try to either reduce or stop your gaming activity?	2.07	0.99	0.79
3. Do you feel the need to spend increasing amount of time engaged gaming in order to achieve satisfaction or pleasure?	2.33	1.07	0.75
4. Do you systematically fail when trying to control or cease your gaming activity?	1.96	0.99	0.79
5. Have you lost interests in previous hobbies and other entertainment activities as a result of your engagement with the game?	2.31	1.14	0.71
6. Have you continued your gaming activity despite knowing it was causing problems between you and other people?	2.05	1.13	0.72
7. Have you deceived any of your family members, therapists or others because the amount of your gaming activity?	1.55	0.93	0.69
8. Do you play in order to temporarily escape or relieve a negative mood (e.g., helplessness, guilt, anxiety)?	3.00	1.20	0.46
9. Have you jeopardized or lost an important relationship, job or an educational or career opportunity because of your gaming activity?	1.44	0.86	0.74

For the IGDS9-SF, the unidimensional model structure had an acceptable fit to the data: χ^2^ = 71.223, CFI = 0.952, TLI = 0.954, RMSEA = 0.064, and SRMR = 0.060. All of the factor loadings were statistically significant; however, Item 8 (escape) had a factor loading below 0.50 (see the lower panel of Table 1). In addition, the mean score of Item 8 was notably higher than the rest of the items.

#### Criterion and Concurrent Validity

**Figure 1:**
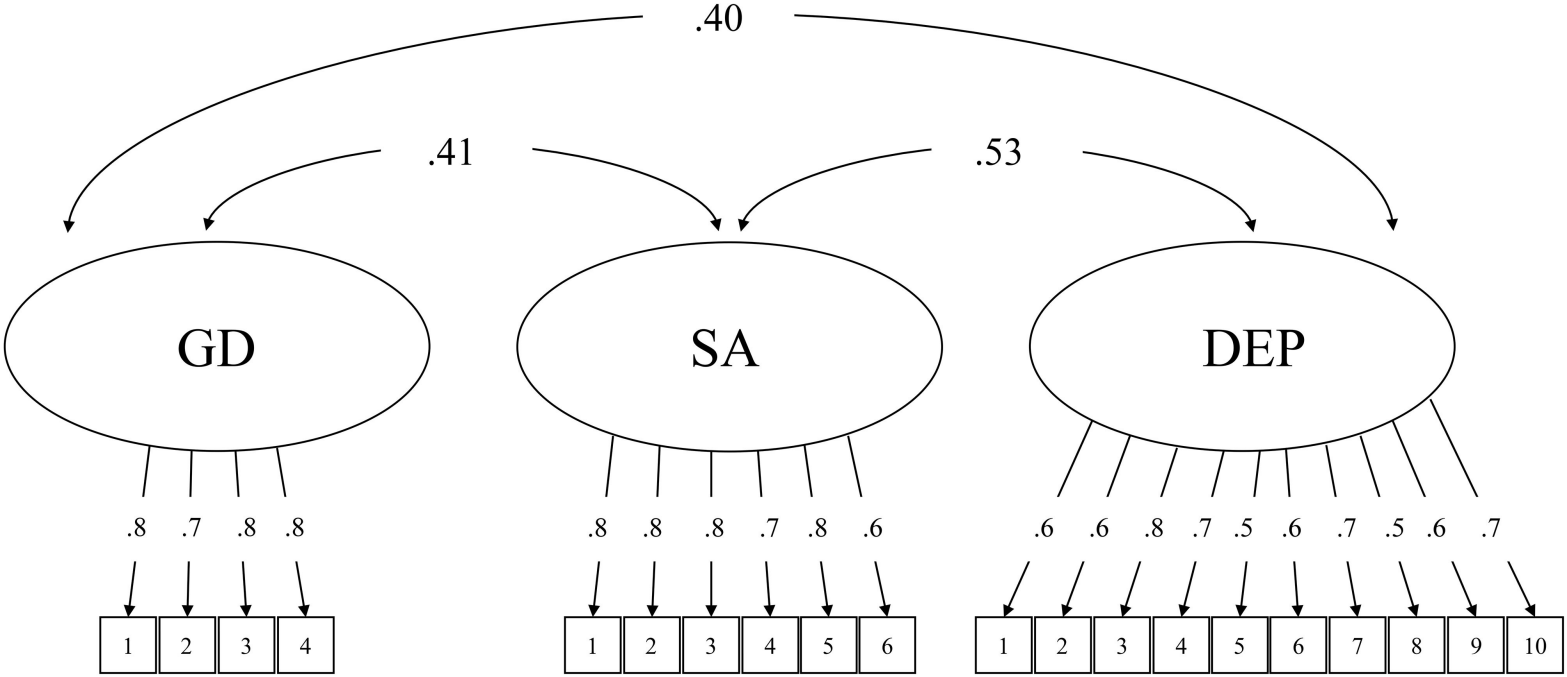
Confirmatory factor analytical model testing the criterion validity of Gaming Disorder Test. GD, gaming disorder (assessed by GDT); SA, social anxiety symptoms; DEP, depressive symptoms. The figures inside the boxes represent the item numbers of the respective measures. All parameters are standardized.

**Figure 2:**
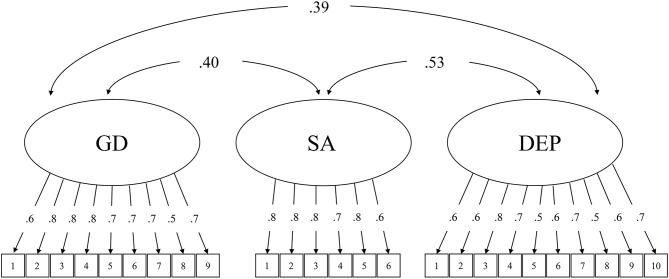
Confirmatory factor analytical model testing the criterion validity of nine-item Internet Gaming Disorder Scale-Short Form. GD, gaming disorder (assessed by IGDS9-SF); SA, social anxiety symptoms; DEP, depressive symptoms. The figures inside the boxes represent the item numbers of the respective measures. All parameters are standardized.

The criterion validity was first tested with correlation analysis. Both the GDT and IGDS9-SF had significant positive associations with social anxiety symptoms (GDT: *r* = 0.41; IGDS9-SF: *r* = 0.40) and depressive symptoms (GDT: *r* = 0.40; IGDS9-SF: *r* = 0.39). To address potential measurement errors, a CFA model was then constructed to further examine the criterion validity of the GDT and IGDS9-SF, respectively (see Figures 1, 2). Both models included three latent variables: gaming disorder, social anxiety symptoms, and depressive symptoms. The results were consistent with the correlation analysis. For the GDT, the model fit indices indicated a good fit with the data: χ^2^ = 362.096, CFI = 0.976, TLI = 0.960, RMSEA = 0.054, and SRMR = 0.062. For the IGDS9-SF, the model fit indices indicated an acceptable fit with the data: χ^2^ = 662.870, CFI = 0.958, TLI = 0.952, RMSEA = 0.060, and SRMR = 0.070.

#### Measurement Invariance

To evaluate measurement invariance, we performed multigroup CFA (16) to assess their measurement and scaling properties across three sample characteristics: gender, age, and employment status. This analysis involves evaluating progressively more constrained models to establish invariance on three levels. At the initial level, configural invariance analysis evaluates whether the number of factors and patterns of free factor loadings are equal across different demographic groups. Specifically, the configural invariance was tested by constraining the unidimensional factor structure to be invariant, but freely estimating the factor loadings and item intercepts. At the next level, metric invariance analysis imposes additional constraints that require the item factor loadings to be equal across demographic groups. At the final level, scalar invariance analysis holds the item intercepts on the latent variables to be equal across groups. When examining the differences among the increasingly more constrained models, relative changes in not only χ^2^ but also CFI and RMSEA were examined, because the chi-square difference test has been found oversensitive in measurement invariance analysis. A change ≤-0.01 in the CFI and a change greater than or equal to 0.015 in the RMSEA were chosen as the cut-offs for non-invariance [see (16, 42)].

#### GDT

The present findings constituted robust evidence of the GDT's scalar measurement invariance for the demographic characteristics of gender, age, and employment status (see the upper panel of Table 2). The cross-model differences evaluated with CFI (all <0.01), RMSEA (all <0.015), and chi-square differences (all non-significant) consistently indicated the invariance of the GDT with respect to the number of factors, item factor loadings, and item intercepts.

**Table 2:** Measurement invariance for gender, age, and employment status for two gaming disorder measures.

**Variable**	**Model**	***df***	**CFI**	**ΔCFI**	**RMSEA**	**ΔRMSEA**	**χ^2^**	**Δχ^2^**
**Gaming Disorder Test**								
**[GDT; (****2****)]**								
Gender	Configural	4	0.978		0.048		2.636	
	Metric	7	0.976	0.002	0.055	0.007	4.028	1.392
	Scalar	18	0.974	0.002	0.061	0.006	15.523	11.495
Age	Configural	6	0.988		0.052		4.209	
	Metric	12	0.985	0.003	0.057	0.005	5.278	1.069
	Scalar	13	0.980	0.005	0.060	0.003	15.840	11.495
Employment Status	Configural	6	0.978		0.055		3.920	
	Metric	12	0.978	0.000	0.061	0.006	8.696	4.776
	Scalar	34	0.977	0.001	0.066	0.005	24.111	15.415
**Nine-item Internet Gaming Disorder Scale-Short Form**								
**[IGDS9-SF; (****7****)]**								
Gender	Configural	54	0.952		0.076		94.608	
	Metric	62	0.946	0.006	0.078	0.002	102.243	7.635
	Scalar	88	0.940	0.006	0.087	0.009	122.451	20.208
Age	Configural	54	0.941		0.067		87.847	
	Metric	62	0.935	0.006	0.072	0.005	110.878	1.069
	Scalar	88	0.930	0.005	0.080	0.008	121.671	11.495
Employment Status	Configural	81	0.933		0.092		163.226	
	Metric	97	0.922	0.010	0.108	0.016	176.872	27.782^*^
	Partial	107	0.932		0.093		175.442	

* *p < 0.05*.

#### IGDS9-SF

Measurement invariance analysis showed the IGDS9-SF to have scalar invariance for the characteristics of gender and age (see the lower panel of Table 2), with such properties supported by the cross-model differences evaluated with multiple indices, including CFI (all <0.01), RMSEA (all <0.015), and chi-square differences (all non-significant).

For the characteristic of employment status, only configural invariance was supported by the model fit indices, and non-invariance was identified in the metric invariance level, as indicated by the increase in RMSEA (i.e., 0.016) and significant chi-square differences. We located the non-invariant parameters by examining the modification indices, with the factor loadings of Item 3 (tolerance) and Item 7 (deception) appearing to be non-invariant. Hence, a partial invariance model with the two non-invariant factor loadings left unconstrained was constructed. This model revealed an adequate and comparable model fit to that of the configural invariance model.

### Incremental Validity

Hierarchical multiple regression analysis was performed to examine the additional contribution of the newly developed GDT over the existing widely used IGDS9-SF to explaining social anxiety symptoms, depressive symptoms, and gaming time. Following the conventional practice of incremental validity analysis, only the constructs identified as significantly associated with the two gaming disorder measures were included in the multiple regression analysis (43). Hence, prior to incremental validity testing, bivariate zero-order correlation analysis was conducted to check whether the proposed constructs should be included in this particular analysis.

#### Social Anxiety and Depressive Symptoms

As shown in Table 3, after controlling for the existing IGDS9-SF measure, the newer GDT measure was unable to explain a significant proportion of the additional variance for social anxiety and depressive symptoms. Thus, the results failed to support the incremental validity of the GDT for predicting these two mental health-related criterion measures.

**Table 3:** Hierarchical multiple regression for evaluating incremental validity of Gaming Disorder Test.

**Gaming type**	**Variable**	***B***	***SE(B)***	**β**	***R*^**2**^**	**Δ*R*^**2**^**
Social anxiety	Step 1				0.160	
	IGDS9-SF	0.337	0.038	0.404		
	Step 2				0.164	0.004
	IGDS9-SF	0.149	0.068	0.201		
	GDT	0.172	0.054	0.218		
Depression	Step 1				0.153	
	IGDS9-SF	0.527	0.062	0.391		
	Step 2				0.157	0.004
	IGDS9-SF	0.256	0.099	0.199		
	GDT	0.262	0.087	0.217		
General gaming time	Step 1				0.053	
	IGDS9-SF	5.091	1.217	0.230		
	Step 2				0.067	0.014^*^
	IGDS9-SF	1.479	2.038	0.067		
	GDT	3.643	1.657	0.202		
Online gaming time	Step 1				0.036	
	IGDS9-SF	3.582	1.046	0.189		
	Step 2				0.057	0.021^*^
	IGDS9-SF	−0.150	1.751	−0.001		
	GDT	3.763	1.430	0.244		

* *p < 0.01*.

#### Gaming Time

Both the GDT (*r* = 0.30) and IGDS9-SF (*r* = 0.25) had significant positive associations with weekly general gaming time. As shown in Table 3, the GDT accounted for a significantly higher proportion of the variance after controlling for the IGDS9-SF. Similar to weekly general gaming time, both the GDT (*r* = 0.29) and IGDS9-SF (*r* = 0.22) were positively associated with weekly online gaming time. Hierarchical multiple regression analysis revealed that after controlling for the IGDS9-SF, the GDT accounted for a significant proportion of the additional variance in this variable. Taken together, these results indicate the incremental validity of the GDT over the IGDS9-SF in explaining both weekly general and weekly online gaming time.

## Discussion

We conducted a psychometric investigation of the GDT developed based on the ICD-11 framework (2) and the IGDS9-SF developed based on the DSM-5 framework (7). For the GDT, the present findings show that it is a reliable measure. The findings also provide support for its structural validity in terms of the excellent model fit across all fit indices, indicating that the unidimensional structure as described in the ICD-11 framework reflects the optimal factor structure for the construct of gaming disorder. This factor structure not only coincides with that proposed in the initial development of GDT (2), but also corroborates with various past attempts to develop and validate measures derived from the DSM-5 framework [e.g., IGDT-10 by Király et al. (44); CIGDS by Sigerson et al. (20)].

For the IGDS9-SF, it is also found to be reliable. In addition, the unidimensionality of such measure has also been supported by most of the fit indices; but Item 8 of this measure— “*Do you play in order to temporarily escape or relieve a negative mood (e.g., helplessness, guilt, anxiety)*?—had a particularly weak factor loading. Moreover, the average score of this item is found considerably higher than the rest of the measure in the present study. It is noteworthy that using games to “escape” has been frequently investigated as a type of gaming motivation, namely, the escapism motivation [e.g., (45, 46)]. Studies have shown that escapism motivation is reported by not only players with abundant gaming disorder symptoms [e.g., (47, 48)] but also those who do not endorse symptoms of such disorder [see review by Lee (49)]. Hence, our findings imply that this particular IGDS9-SF item may not be sufficiently sensitive to differentiate between individuals reporting numerous gaming disorder symptoms and those reporting few such symptoms.

Second, evidence of configural, metric, and scalar invariance was found for GDT concerning all three demographic characteristics (i.e., gender, age, and employment status), whereas for the IGDS9-SF, such evidence was found for gender and age alone, with the results only partially supporting metric invariance for employment status. More specifically, the findings revealed that Item 3 (tolerance) and Item 7 (deception) had weaker factor loadings in the student sample than in the employed sample.

Such variations may be attributable to the different interpretations of these items regarding in-game investment. Conventionally, gaming time has been conceptualized as the primary form of such investment [e.g., (50, 51)]. For instance, both Item 3 (tolerance) and Item 7 (deception) were evaluated based on the “amount of time” spent on gaming. However, some scholars maintain that in-game monetary expenditure should also be acknowledged as a form of investment (52–54). Thus, it is important to consider how monetary and non-monetary (e.g., time) investment may differentially influence distinct groups of players. For instance, long gaming time has been identified as a concern for employed players, as it can interfere with their work performance and even result in disciplinary action [e.g., (55, 56)]. In contrast, recent findings indicate that games encouraging high in-game expenditure tend to jeopardize adolescents, who are not financially independent, and may thus have to deceive family members or friends to cover that expenditure (57). Hence, the undesirable outcomes associated with a greater amount of time spent on gaming may be perceived differently by student players and employed players.

Lastly, our analysis produced mixed findings concerning the incremental validity of the GDT over the IGDS9-SF. For instance, the results showed the GDT to have incremental validity over the IGDS9-SF in predicting both general and online gaming time, but no such validity was found for either depressive or social anxiety symptoms. Such incremental validity concerning gaming time may reflect potential differences between the more concise ICD-11 criteria and more comprehensive DSM-5 criteria. In addition to the criterion of “escape,” several additional criteria in the DSM-5 framework, including withdrawal and tolerance symptoms, have also been scrutinized concerning their relevance to gaming behavior [e.g., (50, 58)]. A recent review (59) maintained that gaming disorder may arise without the presence of withdrawal symptoms, and the current conceptualization of tolerance symptoms has also been criticized for its direct adaptation from substance addiction criteria without considering the nature of gaming activity (51). Thus, such criteria may partially weaken the explanatory power of the IGDS9-SF for time spent on gaming.

### Implications for Future Research and Practice

The findings of this study have several implications for future research on gaming disorder. As the literature contains a number of gaming disorder measures developed under the DSM-5 framework (e.g., IGDS9-SF, IGDT-10), whereas a more recent measure was developed under the new ICD-11 framework (i.e., GDT), researchers may wonder which should be the most appropriate measure for their studies. As a guide for researchers, our study identifies two major issues to consider when deciding which measure to use to assess gaming disorder.

A major factor to consider is the length of a gaming disorder measure (6). As impulsivity has frequently been identified as a risk factor for such disorder [e.g., (60, 61)], brief assessment tools are preferable for use among individuals with high levels of impulsivity (2). Moreover, brief measures are also preferred for use in online surveys, because a shorter survey length has been associated with lower drop-out rates (62). Hence, the shorter GDT, at just four items, may be a more suitable choice to meet these purposes. Compared to the IGDS9-SF whose length is twice longer, the GDT provides an equally good or even better explanation of the data, as indicated by its incremental validity over the IGDS9-SF in accounting for variances in gaming time, as well as its scalar invariance property concerning employment status that is not found for the IGDS9-SF. Hence, researchers requiring methods with a short protocol (e.g., telephone survey) will find the GDT to be a brief, valid assessment tool that can be easily administered to a heterogeneous sample.

Another major factor to consider is the comprehensiveness of a measure, that is, whether the measure sufficiently examines all important domains of gaming disorder (63). Compared to the GDT, the IGDS9-SF offers a more comprehensive examination of gaming disorder based on the nine criteria. The comprehensive scope of the IGDS9-SF makes it potentially more appropriate for use in studies examining multidimensional gaming-related constructs that evaluate various aspects of gaming (e.g., gaming motivation, player identification). However, in our psychometric analyses, we identified several IGDS9-SF items that require further scrutiny. For example, similar to previous studies [e.g., (26, 64)], we found the item assessing the “escape” criteria to have poor discriminatory power. Thus, future inquiries should evaluate whether this item is a precise indicator of gaming disorder, or whether it simply reflects a high, but not problematic, level of engagement in gaming. Such a conceptual distinction is necessary because excessive gaming and high engagement in gaming have been found to represent diverse constructs, with the former explaining a large portion of variance in socially undesirable personality characteristics (e.g., introversion, neuroticism) while no such findings are yielded for the latter (65). It is also noteworthy that the two IGDS9-SF items concerning tolerance and deception yielded a poorer estimation of gaming disorder severity among student players than employed players. Hence, researchers should take respondents' employment status into consideration when using the IGDS9-SF in future studies.

A third major factor to consider is item wording. Although several items appear in both the GDT and IGDS9-SF, it is necessary to scrutinize the differences regarding the wording of this common set of items. For instance, one item included in the IGDS9-SF has stronger emphasis on the interpersonal difficulties of players compared to that of the GDT. Specifically, Item 2 of the GDT reads: “*I have continued gaming despite the occurrence of negative consequences*,” whereas the corresponding item in the IGDS9-SF (Item 6) reads: “*Have you continued your gaming activity despite knowing it was causing problems between you and other people?*” Hence, recent experiences of interpersonal problems are necessary for the item to be endorsed for the IGDS9-SF, but the item of GDT is endorsed for respondents who have recent experiences of problems in any life domains (e.g., work/studies, health, and interpersonal relations). However, it is likely that the severity of gaming disorder for players with a sparse social network may be underestimated because they tend to have fewer undesirable interpersonal experiences, compared to their counterparts having a dense social network in which interpersonal conflicts are more likely to occur [e.g., (66, 67)]. Thus, researchers should also consider the implication of this wording choice when selecting measures for assessing gaming disorder.

Furthermore, the present findings also have implications for practitioners. Gaming disorder has recently been classified as one of the “disorders due to addictive behaviors” in the ICD-11. Healthcare professionals may utilize the diagnostic criteria and codes outlined in either the ICD-11 or DSM-5 for assessing symptoms of this emergent psychological problem. The present study provides empirical support for both the GDT and IGDS9-SF as valid measures for symptom assessment. However, the IGDS9-SF is preferred for screening clients who are at-risk for gaming disorder, because an optimal cut-off value has been identified for this measure with high sensitivity and specificity for distinguishing between gamers clinically diagnosed as having gaming disorder and those without this diagnosis (68). As GDT is a newer measure of gaming disorder, sensitivity, and specificity tests should be conducted among clinical samples to obtain a clinically useful cut-off for screening at-risk clients.

### Research Caveats

Several potential limitations of this study should be noted. First, the study evaluated the utility of both the GDT and IGDS9-SF for use with non-clinical samples. Accordingly, its findings may not be generalizable to clinical populations, such as individuals diagnosed with clinical depression. Future studies should further validate the GDT with clinical samples to allow comparisons to be made between clinical and non-clinical samples exhibiting gaming disorder symptoms.

Second, our participants were recruited primarily from North America and Europe, and hence the results may not be generalizable to other cultural regions, including East Asia and South America. Given that gaming disorder is a global concern (1), further effort should be made to translate the new and promising GDT into various languages for psychometric validation. Previous multinational comparisons have identified considerable differences in thinking and behavior among the inhabitants of diverse cultural regions (69), and the measurement invariance of cultural region should be established to allow more extensive cross-cultural comparisons of the prevalence of gaming disorder.

Lastly, the present study employs the IGDS9-SF as the assessment tool developed under the DSM-5 framework. It is important to acknowledge that there are at least six other assessment tools that have been developed based on this framework, and a recent review has documented notable differences among these instruments regarding the number of items (ranging from 9 and 27 items), response format, and psychometric properties (6). Hence, although we aimed to evaluate and compare the psychometric properties of the GDT (derived from the ICD-11 framework) with the IGDS9-SF (derived from the DSM-5 framework), researchers should be cautious in generalizing the present findings to other assessment tools developed under the DSM-5 framework.

## Conclusion

This study provides the first psychometric comparison of the new GDT and the popular IGDS9-SF. Both measures are found to be valid, reliable tools to assess gaming disorder; however, some notable differences have been identified regarding the properties of measurement invariance. In addition, the GDT demonstrates incremental validity over the IGDS9-SF in explaining gaming time. Based on these findings, we advise researchers to consider the factors of brevity and comprehensiveness when choosing an appropriate measure of gaming disorder.

## Data Availability Statement

The raw data supporting the conclusions of this article will be made available by the authors, without undue reservation.

## Ethics Statement

The studies involving human participants were reviewed and approved by University of Hong Kong's Human Research Ethics Committee. The participants provided online informed consent to participate in this study.

## Author Contributions

The study was designed by authors CC and H-YW. H-YW coordinated the data collection process, performed all the statistical analyses, and completed the first draft of the manuscript. CC contributed to the editing and revision of the manuscript. All authors contributed to the article and approved the submitted version.

## Conflict of Interest

The authors declare that the research was conducted in the absence of any commercial or financial relationships that could be construed as a potential conflict of interest.

## Footnotes

^1^The methods for testing various types of measurement invariance were commonly adopted in previous studies [e.g., (20–23)].
